# Knocking out central metabolism genes to identify new targets and alternating substrates to improve lipid synthesis in *Y. lipolytica*

**DOI:** 10.3389/fbioe.2023.1098116

**Published:** 2023-01-13

**Authors:** Jiang Zhu, Yang Gu, Yijing Yan, Jingbo Ma, Xiaoman Sun, Peng Xu

**Affiliations:** ^1^ School of Food Science and Pharmaceutical Engineering, Nanjing Normal University, Nanjing, China; ^2^ Department of Chemical, Biochemical and Environmental Engineering, University of MD, Baltimore County, Baltimore, MD, United States; ^3^ College of Biological and Pharmaceutical Engineering, West Anhui University, Lu’an, Anhui, China; ^4^ Department of Chemical Engineering, Guangdong Technion-Israel Institute of Technology (GTIIT), Shantou, Guangdong, China; ^5^ The Wolfson Department of Chemical Engineering, Technion-Israel Institute of Technology, Haifa, Israel

**Keywords:** *Yarrowia lipolytica*, lipogenesis, central metabolism, lipid synthesis, metabolic engineering

## Abstract

**Introduction:** Systematic gene knockout studies may offer us novel insights on cell metabolism and physiology. Specifically, the lipid accumulation mechanism at the molecular or cellular level is yet to be determined in the oleaginous yeast *Y. lipolytica*.

**Methods:** Herein, we established ten engineered strains with the knockout of important genes involving in central carbon metabolism, NADPH generation, and fatty acid biosynthetic pathways.

**Results:** Our result showed that NADPH sources for lipogenesis include the OxPP pathway, POM cycle, and a trans-mitochondrial isocitrate-α-oxoglutarate NADPH shuttle in *Y. lipolytica*. Moreover, we found that knockout of mitochondrial NAD^+^ isocitrate dehydrogenase IDH2 and overexpression of cytosolic NADP^+^ isocitrate dehydrogenase IDP2 could facilitate lipid synthesis. Besides, we also demonstrated that acetate is a more favorable carbon source for lipid synthesis when glycolysis step is impaired, indicating the evolutionary robustness of *Y. lipolytica*.

**Discussion:** This systematic investigation of gene deletions and overexpression across various lipogenic pathways would help us better understand lipogenesis and engineer yeast factories to upgrade the lipid biomanufacturing platform.

## 1 Instruction

Currently, concerns about fossil fuel sustainability have driven researchers to develop renewable lipid-derived biofuels as an alternative (Gu et al., 2018; Lazar et al., 2018). Lipid synthesis using oleaginous yeasts, owing to their unique metabolism, can achieve high lipid titers, yields and productivity, thus creating the potential for cost-effective industrial-scale operations (Ma et al., 2020). Specifically, *Yarrowia lipolytica* is an oleaginous yeast with GRAS (generally regarded as safe) status and has several significant attributes, such as a broad substrate spectrum, relatively well-defined genome annotations, diversified genetic manipulation methods, and abundant pool size of metabolic precursors, including acetyl-CoA, malonyl-CoA, and tricarboxylic acid (TCA) cycle intermediates (Abdel-Mawgoud et al., 2018; Larroude et al., 2018; Markham and Alper, 2018). Therefore, this yeast has emerged as a popular non-model organism in the lipid biomanufacturing field (Liu et al., 2015; Wong et al., 2017; Liu et al., 2019a; Yang et al., 2019).

In *Y. lipolytica*, extracellular carbon feedstock, such as glucose, is firstly internalized and converted into cytosolic pyruvate by glycolytic pathway (Qiao et al., 2017). Then, pyruvate is transported into mitochondria to synthesize mitochondrial citrate. Next, citrate is excreted to the cytoplasm by mitochondrial citrate carrier YlYhm2p (Yuzbasheva et al., 2019) and further cleaved to acetyl-CoA for lipid synthesis by cytosolic ATP-citrate lyases (Qiao et al., 2017). However, lipids are the highly reduced metabolites that need to consume the NADPHs to reduce acetyl groups (CH_3_-CO-) to alkyl groups (-CH_2_-CH_2_-) for growing fatty acid carbon backbone (Wasylenko et al., 2015; Xu et al., 2017; Xue et al., 2017). Currently, optimizing the intracellular metabolic network of *Y. lipolytica* has become a trend to improve lipids production by metabolic engineering and synthetic biology methods (Qiao et al., 2015; Liu et al., 2016; Xu et al., 2016; Abdel-Mawgoud et al., 2018). For example, Lazar et al. knocked out POX1-6 (peroxidase) and TGL4 (triglyceride lipase) with overexpressing GDP1 (glycerol triphosphate dehydrogenase) and DGA2 (diacylglycerol acyltransferase) in *Y. lipolytica*, improving the titer of lipids to 34 g/L (Dulermo and Nicaud, 2011). Qiao et al. optimized the supply of NADPH, which maximized the utilization of electrons from alternative NADPH pathways to increase substrate-to-lipid yields, leading to a 25% improvement over the starting strain (Qiao et al., 2017). Notwithstanding, much effort has been investigated to improve lipid accumulation, such as deletion of degradation pathways, overexpression of lipid synthesis pathways, eliminating of regulatory bottlenecks, and strengthening the supply of precursor, such as acetyl-CoA and NADPH (Groenewald et al., 2014; Silverman et al., 2016; Liu et al., 2019b). However, the influences of the important gene deficiency on lipid accumulation, such as carbon feedstock degradation and NADPH metabolism, have not been explored in *Y. lipolytica*.

Herein, we systematically investigated the influence of deleting important lipogenesis genes on lipid synthesis and cell growth, involving in carbon metabolism, NADPH metabolism, and fatty acid biosynthetic pathways. Specifically, we demonstrated that the sources of NADPH for lipogenesis include the OxPPP pathway, pyruvate-oxaloacetate-malate (POM) cycle, and isocitrate-2-oxoglutarate shuttle in *Y. lipolytica*. Moreover, the knockout of mitochondrial NAD^+^ isocitrate dehydrogenase IDH2 and overexpression of cytosolic NADP^+^ isocitrate dehydrogenase IDP2 could facilitate lipid synthesis. Besides, we also concluded that acetate is a favorable carbon source for producing lipid in *Y. lipolytica*. This study may help us better understand lipogenesis mechanisms, and the insights obtained here will guide us to engineer efficient cell factories for oleochemical production.

## 2 Materials and methods

### 2.1 Strains, plasmid, primers, and chemicals

The strain po1fk obtained in our previous work was chosen as the starting strain in this study (Gu et al., 2020a). Moreover, strains, plasmids and primers have been listed in Table1 and Supplementary Table S1, respectively. Chemicals, including methyl tridecanoic acid and triglyceryl heptadecanoic acid were purchased from Sigma-Aldrich.

**TABLE 1:** Strains and plasmids used in this study.

Names	Characteristics	References
Strains		
po1fk	Wild-type strain W29 (ATCC20460) derivate; W29 *ΔmatA Δxpr*2-332 *Δaxp*-2 *Δleu*2-270 pBR platform, deletion of gene *ura* and *ku70*; po1g *Δura*3 *Δku70::loxP*	Gu et al. (2020a); Gu et al. (2020b)
po1fk_*ylPFK*	po1fk derivate; Further deletion of gene *ylPFK*; po1fk *ΔylPFK::hygr*	This study
po1fk_*ylPYK*	po1fk derivate; Further deletion of gene *ylPYK*; po1fk *ΔylPYK::hygr*	This study
po1fk_*ylACO1*	po1fk derivate; Further deletion of gene *ylAC O 1*; po1fk *ΔylAC O 1::hygr*	This study
po1fk_*ylMAE1*	po1fk derivate; Further deletion of gene *ylMAE1*; po1fk *ΔylMAE1::hygr*	This study
po1fk_*ylIDH2*	po1fk derivate; Further deletion of gene *ylIDH2*; po1fk *ΔylIDH2::hygr*	This study
po1fk_*ylIDP2*	po1fk derivate; Further deletion of gene *ylIDP2*; po1fk *ΔylIDP2::hygr*	This study
po1fk_*ylZWF1*	po1fk derivate; Further deletion of gene *ylZWF1*; po1fk *ΔylZWF1::hygr*	This study
po1fk_*ylPYC1*	po1fk derivate; Further deletion of gene *ylPYC1*; po1fk *ΔylPYC1::hygr*	This study
po1fk_*ylFAA1*	po1fk derivate; Further deletion of gene *ylFAA1*; po1fk *ΔylFAA1::hygr*	This study
po1fk_*ylSNF1*	po1fk derivate; Further deletion of gene *ylFAA1*; po1fk *ΔylSNF1::hygr*	This study
po1fk pYLXP’-*ylPFK*	po1fk derivate; Further expression of gene *ylPFK*	This study
po1fk pYLXP’-*ylPYK*	po1fk derivate; Further expression of gene *ylPYK*	This study
po1fk pYLXP’-*ylACO1*	po1fk derivate; Further expression of gene *ylAC O 1*	This study
po1fk pYLXP’-*ylMAE1*	po1fk derivate; Further expression of gene *ylMAE1*	This study
po1fk pYLXP’-*ylIDH2*	po1fk derivate; Further expression of gene *ylIDH2*	This study
po1fk pYLXP’-*ylIDP2*	po1fk derivate; Further expression of gene *ylIDP2*	This study
po1fk pYLXP’-*ylZWF1*	po1fk derivate; Further expression of gene *ylZWF1*	This study
po1fk pYLXP’-*ylPYC1*	po1fk derivate; Further expression of gene *ylPYC1*	This study
po1fk pYLXP’-*ylFAA1*	po1fk derivate; Further expression of gene *ylFAA1*	This study
po1fk pYLXP’-*ylSNF1*	po1fk derivate; Further expression of gene *ylFAA1*	This study
Plasmids		
pYLXP’-loxP-Hygr	pYLXP’ containing the *loxP-hygr-loxP* cassette	Gu et al. (2020a); Gu et al. (2020b)
pHyloxP-*ylPFK*	pYLXP’*-loxP-hygr* containing gene *ylPFK deletion cassette*	This study
pHyloxP-*ylPYK*	pYLXP’*-loxP-hygr* containing gene *ylPYK deletion cassette*	This study
pHyloxP-*ylACO1*	pYLXP’*-loxP-hygr* containing gene *ylAC O 1 deletion cassette*	This study
pHyloxP-*ylPYC1*	pYLXP’*-loxP-hygr* containing gene *ylPYC1 deletion cassette*	This study
pHyloxP-*ylMAE1*	pYLXP’*-loxP-hygr* containing gene *ylMAE1 deletion cassette*	This study
pHyloxP-*ylIDH2*	pYLXP’*-loxP-hygr* containing gene *ylIDH2 deletion cassette*	This study
pHyloxP-*ylIDP2*	pYLXP’*-loxP-hygr* containing gene *ylIDP2 deletion cassette*	This study
pHyloxP-*ylZWF1*	pYLXP’*-loxP-hygr* containing gene *ylZWF1 deletion cassette*	This study
pHyloxP-*ylFAA1*	pYLXP’*-loxP-hygr* containing gene *ylFAA1 deletion cassette*	This study
pHyloxP-*ylSNF1*	pYLXP’*-loxP-hygr* containing gene *ylSNF1 deletion cassette*	This study
pYLXP’-*ylPFK*	pYLXP’ containing gene *ylPFK expression cassette*	This study
pYLXP’-*ylPYK*	pYLXP’ containing gene *ylPYK expression cassette*	This study
pYLXP’-*ylACO1*	pYLXP’ containing gene *ylAC O 1 expression cassette*	This study
pYLXP’-*ylPYC1*	pYLXP’ containing gene *ylPYC1 expression cassette*	This study
pYLXP’-*ylMAE1*	pYLXP’ containing gene *ylMAE1 expression cassette*	This study
pYLXP’-*ylIDH2*	pYLXP’ containing gene *ylIDH2 expression cassette*	This study
pYLXP’-*ylIDP2*	pYLXP’ containing gene *ylIDP2 expression cassette*	This study
pYLXP’-*ylZWF1*	pYLXP’ containing gene *ylZWF1 expression cassette*	This study
pYLXP’-*ylFAA1*	pYLXP’ containing gene *ylFAA1 expression cassette*	This study
pYLXP’-*ylSNF1*	pYLXP’ containing gene *ylSNF1 expression cassette*	This study

### 2.2 Gene knockout vectors construction

The marker-free Cre-*loxP* based gene knockout method was used as previously reported (Gu et al., 2020a; Gu et al., 2020b). In this study, constructed gene knockout plasmids included pHyloxP-*ylPFK* (encoding phosphofructose kinase), pHyloxP-*ylPYK* (encoding pyruvate kinase), pHyloxP-*ylPYC1* (encoding pyruvate carboxylase), pHyloxP-*ylMAE1* (encoding malic enzyme), pHyloxP-*ylIDH2* (encoding mitochondrial isocitrate dehydrogenase), pHyloxP-*ylIDP2* (encoding cytosolic isocitrate dehydrogenase), pHyloxP-*ylFAA1* (encoding peroxisomal fatty acyl-CoA ligase), pHyloxP-*ylACO1* (encoding aconitate hydratase), and pHyloxP-*ylSNF1* (encoding AMP-activated protein kinase).

Here, we took the process of constructing the plasmid pHyloxP-*ylPFK* as an example. Firstly, the upstream and downstream sequences (both 1000 bp) of flanking gene *ylPFK* were obtained by the PCR-amplified reactions using primers ylPFK_UpF/ylPFK_UpR and ylPFK_DwF/ylPFK_DwR. Then, pYLXP’-*loxP*-*Hyr* containing *loxP*-*Hyr*-*loxP* cassette was digested by endonucleases *AvrII* and *SalI* to obtain *loxP*-*Hyr*-*loxP* cassette and plasmid backbone of pYLXP’-*loxP*-*Hyr*. Next, the upstream fragment ylPFK_Up, downstream fragments ylPFK_Dw, *loxP*-*Hyr*-*loxP* cassette and pYLXP’-*loxP*-*Hyr* backbone were joined by Gibson Assembly to generate the plasmid pHyloxP-*ylPFK*. The constructed plasmids were sequenced by Sangon Biotech Co., Ltd. (Shanghai, China).

### 2.3 The expression plasmid construction and assembly

The pYLXP’, a YaliBrick plasmid, was used for gene expression (Gu et al., 2020a; Gu et al., 2020b). For example, to construct the recombinant plasmid pYLXP’-ylPFK, pYLXP’ was firstly digested by *SnaBI* and *KpnI*, obtaining the linearized pYLXP’. Then, the linearized pYLXP’ was ligase with the DNA fragment of gene PFK (that was obtained by the PCR-amplified reaction using primers PFK-F and PFK-R) by Gibson assembly method to generate plasmid pYLXP’-ylPFK, which was sequenced by Sangon Biotech Co., Ltd. (Shanghai, China).

### 2.4 Yeast transformation

The standard protocols of *Y. lipolytica* transformation have been reported (Gu et al., 2020b; Lv et al., 2020). In brief, 1-mL cells were harvested from YPD medium (yeast extract 10 g/L, peptone 20 g/L, and glucose 20 g/L) at 24 h. Then, cells were resuspended by using 105 uL transformation solution, containing 90 uL 50% PEG4000, 5 uL lithium acetate (2 M), 5 uL boiled single strand DNA (salmon sperm, denatured), and 5 uL DNA products. Next, the mixture was incubated at 39°C for 1 h before spreading on selected plates, which needed to be vortexed for 15 s every 15 min. In this study, the selected markers, including leucine and hygromycin, were used.

### 2.5 Shake flask cultivations

Shake flask cultivations of genetically modified *Y. lipolytica* were performed in 250 mL flasks with 25 mL fermentation medium at 220 r.p.m. and 30°C for 120 h. For this, 0.8 mL seed solution was inoculated, which was cultured in 14 mL shaking tubes under the conditions of 220 r.p.m. and 30°C for 48 h. One milliliter of fermentation broth was sampled every 24 h for OD_600_, lipid, glucose, and acetate measurements.

Seed culture medium of CSM (the yeast complete synthetic media) contains ammonium sulfate 5.0 g/L, YNB (yeast nitrogen base without ammonium sulfate) 1.7 g/L, glucose 20.0 g/L, CSM-Leu 0.74 g/L, and L-leucine 0.20 g/L. The CSM fermentation medium (C/N = 80) contains ammonium sulfate 1.1 g/L, YNB 1.7 g/L, glucose 40.0 g/L, CSM-Leu 0.74 g/L, and L-leucine 0.20 g/L. Specifically, phosphoric buffer solution (PBS), including 0.2 M Na_2_HPO_4_ and 0.2 M Na_2_HPO_4_, with pH 6.0 was used to replace water to make CSM-Acetate fermentation medium. Besides, CSM-acetate medium (C/N = 80) contains ammonium sulfate 1.1 g/L, YNB 1.7 g/L, acetate 41.0 g/L, CSM-Leu 0.74 g/L, and L-leucine 0.20 g/L.

### 2.6 Quantification of biomass, glucose, citrate and lipid

Cell densities were monitored by measuring the optical density at 600 nm (OD_600_). The OD_600_ value could be converted to dry cell weight (DCW) according to Eq. 1 OD_600_ = 0.35 g/L. The concentrations of glucose and acetate were measured under a flow rate of 0.6 mL/min of the mobile phase (5 mM of H_2_SO_4_) at 40°C by high-performance liquid chromatography (HPLC) equipped with a HPX-87H column (Bio-Rad, Hercules, CA, United States) and a refractive index detector through Shimadzu Prominence HPLC LC-20 A. To determine lipid composition, 4 OD units of cells were directly saponified with 0.5 M sodium methoxide with vortexing at 1,200 rpm for 2 h, and then neutralized with 40 μL 98% sulfuric acid to facilitate transesterification. Next, 400 μL hexane was added to extract fatty acid methyl ester for GC analysis. Moreover, to quantitatively determine lipid titer, 100 μL of 2 g/L of tridecanoate methyl ester and 2 g/L of glyceryl trihepta-decanoate were added as internal standard at the beginning, to benchmark the transesterification and saponification efficiency, respectively. Triplicate samples were taken and results were reported as average ± standard deviations. Lipid samples were taken at 120 h of shaking incubation. The method of GC analysis is consistent with previously reported method (Xu et al., 2016).

## 3 Results

### 3.1 Selecting the important genes for investigating the lipogenesis metabolism in *Y. lipolytica*

Noticeably, cytosolic acetyl-CoA is a direct precursor for lipid synthesis (Qiao et al., 2017), which is carboxylated to malonyl-CoA, further transacetylated to malonyl-ACPs, the true precursor to extending the growing chain of fatty acids, catalyzed by fatty acid synthases (FAS1 and FAS2). Thus, in acetyl-CoA metabolism, we chose three genes for investigation, including *ylPFK* (*YALI0D16357g*, encoding 6-phosphofructokinase), *ylPYK* (*YALI0F09185g*, encoding pyruvate kinase), and *ylAC O 1* (*YALI0D09361g*, encoding aconitate hydratase), and further constructed three strain po1fk_*ylPFK,* po1fk_*ylPYK*, and po1fk_*ylACO1*.

**FIGURE 1:**
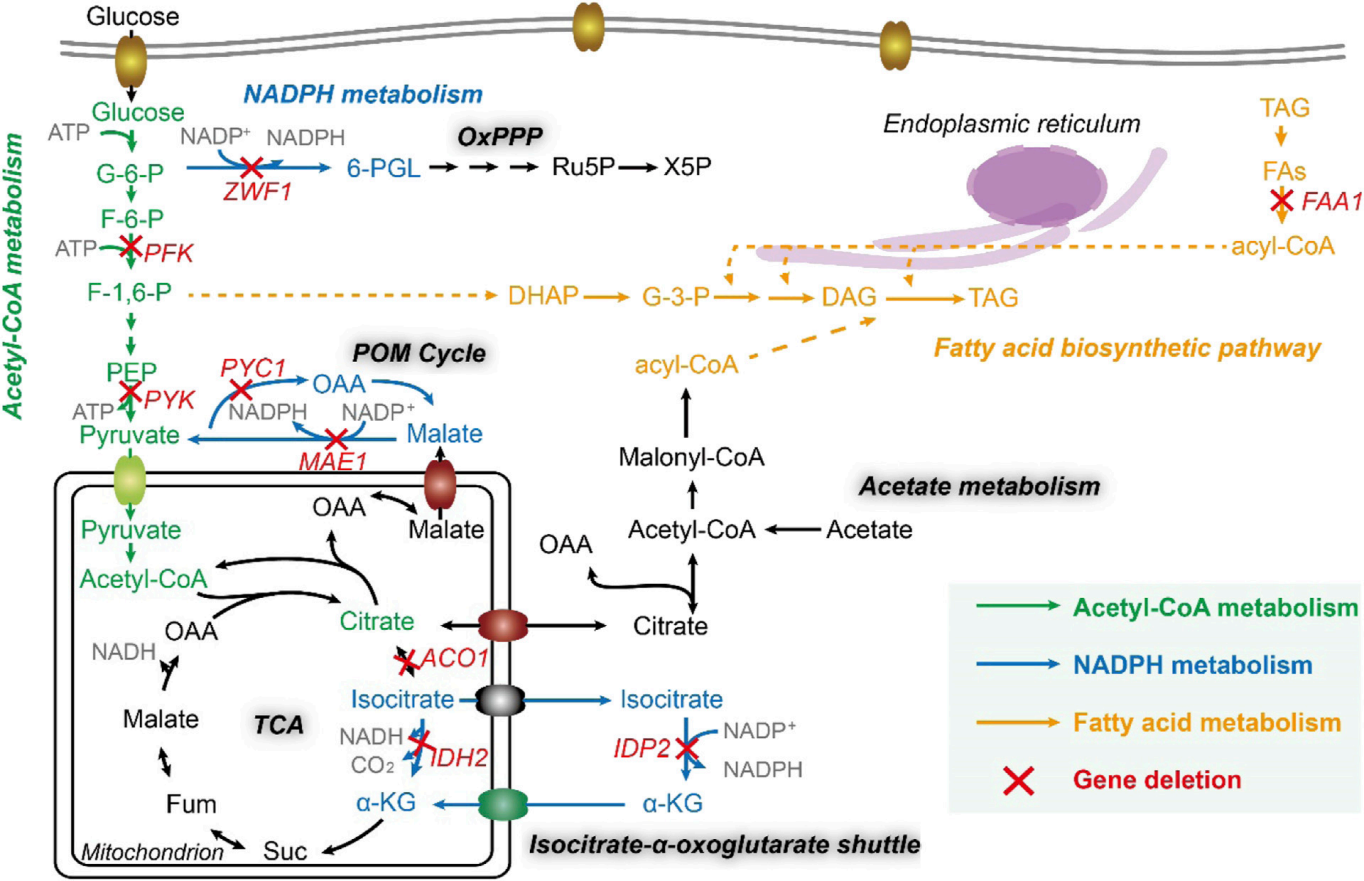
The fatty acid metabolic network of Y. lipolytica. FAA1, fatty acyl-CoA synthase; ZWF1, glucose-6-phosphate dehydrogenase; PFK, 6-phosphofructokinase; PYK, pyruvate kinase; PYC1, pyruvate carboxylase; MAE1, malic enzyme; ACO1, Aconitase; IDH2, mitochondrial isocitrate dehydrogenase; IDP2, cytosolic NADP-specific isocitrate dehydrogenase; G-6-P, Glucose-6-phosphate; F-6-P, fructose-6-phosphate; F-1,6-P, fructose-1,6-diphosphate; PEP, phosphoenolpyruvic acid; OAA, oxaloacetic acid; Fum, fumaric acid; Suc, succinyl coenzyme A; α-KG, 2-oxoglutarate; 6-PGL, 6-phosphogluconolactone; Ru5P, ribulose-5-phosphate; X5P, xylulose-5-phosphate; DHAP, dihydroxyacetone phosphate; G-3-P, glyceraldehyde-3-phosphate; DAG, diacylglycerol; TAG, triacylglycerol; FFA, free fatty acid; PL, phospholipid; Glx, glutamine; QH2, coenzyme QH2.

On the other hand, acetyl groups (CH_3_-CO-) originating from acetyl-CoA is reduced to alkyl groups (-CH_2_-CH_2_-) when growing fatty acid carbon backbone, which needs to consume the reducing equivalent NADPH (Liu et al., 2019a). It has been reported that the sources of cytosolic NADPH include NADP^+^-dependent isocitrate dehydrogenase, the OxPP pathway, and POM cycle (Silverman et al., 2016), related to malate dehydrogenase (encoded by gene *ylMAE1*, *YALI0E18634g*), mitochondrial isocitrate dehydrogenase (encoded by gene *ylIDH2*, *YALI0D06303g*), cytosolic isocitrate dehydrogenase (encoded by gene *ylIDP2*, *YALI0F04095g*), and glucose-6-phosphate dehydrogenase (encoded by gene *ylZWF1*, *YALI0E22649g*). Particularly, the POM cycle (Figure 1) also involves the reductive carboxylation of pyruvate to oxaloacetate by pyruvate carboxylase (encoded by gene *ylPYC1*, *YALI0C24101g*). Thus, these five genes in the NADPH metabolism were deleted to obtained strains po1fk_*ylMAE1,* po1fk_*ylIDH2*, po1fk_*ylIDP2*, po1fk_*ylZWF1*, and po1fk_*ylPYC1*, respectively. Moreover, we also investigated the influences of deleting lipogenesis gene *ylFAA1* (*YALI0D17864g*, long chain fatty acyl-CoA synthetase) on the lipid synthesis (Figure 1), which generated strain po1fk_*ylFAA1*. In addition, the regulator SNF1 (encoded by gene *YALI0D02101g*, carbon catabolite-derepressing protein kinase) was also knocked out, which is a cellular carbon metabolism regulator in *Y. lipolytica* to control the transition from growth phase to oleaginous production phase (Lazar et al., 2018), obtaining po1fk_*ylSNF1*.

### 3.2 Characterization of engineered *Y. lipolytica* strains under the nitrogen-limited shaking cultivation

**FIGURE 2:**
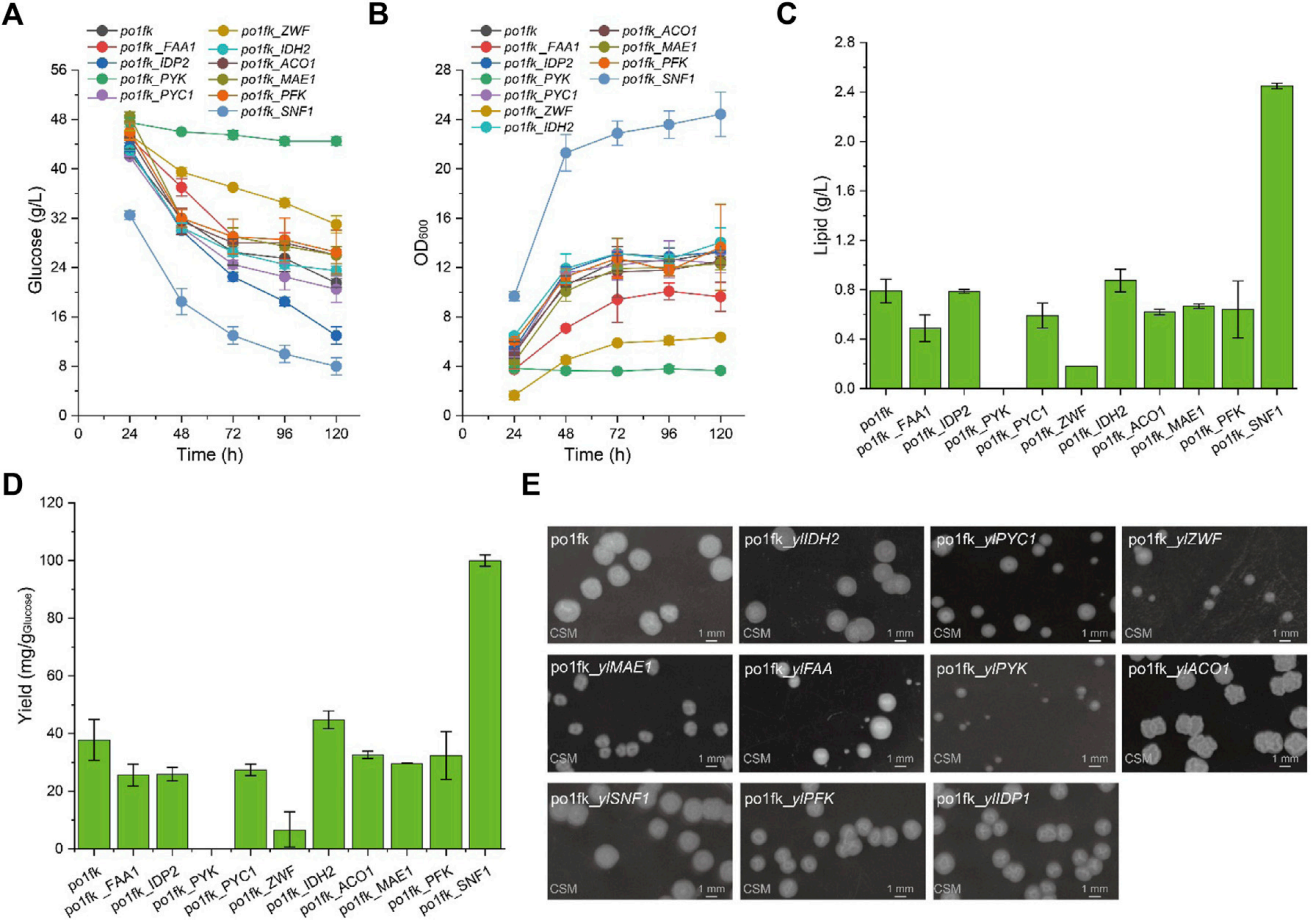
Time profiles of cell growth, glucose consumption, lipid titer and lipid yield in the CSM-leu medium and the morphology analysis of gene knockout strains. **(A)** Glucose consumption of po1fk, po1fk_ylPYK, po1fk_ylACO1, po1fk_ylPFK, po1fk_ylSNF1, po1fk_ylIDP2, po1fk_ylZWF, po1fk_ylIDH2, po1fk_ylMAE, po1fk_ylPYC1, and po1fk_ylFAA; **(B)** Cell growth of po1fk, po1fk_ylPYK, po1fk_ylACO1, po1fk_ylPFK, po1fk_ylSNF1, po1fk_ylIDP2, po1fk_ylZWF, po1fk_ylIDH2, po1fk_ylMAE, po1fk_ylPYC1, and po1fk_ylFAA; **(C)** Lipid titer of po1fk, po1fk_ylPYK, po1fk_ylACO1, po1fk_ylPFK, po1fk_ylSNF1, po1fk_ylIDP2, po1fk_ylZWF, po1fk_ylIDH2, po1fk_ylMAE, po1fk_ylPYC1, and po1fk_ylFAA; **(D)** Lipid yield of po1fk, po1fk_ylPYK, po1fk_ylACO1, po1fk_ylPFK, po1fk_ylSNF1, po1fk_ylIDP2, po1fk_ylZWF, po1fk_ylIDH2, po1fk_ylMAE, po1fk_ylPYC1, and po1fk_ylFAA; **(E)** the morphology analysis of gene knockout strains. For analyzing the morphology, cells were harvested from CSM-leu medium at 48 h and washed twice using .9% of NaCl solution. Then, cells were re-suspended and diluted to an appropriate concentration and spread on the CSM plates, cultivating at 30°C for 96 h.

To confirm whether cell growth and lipid levels of engineered *Y. lipolytica* strains were changed, we performed the nitrogen-limited shaking cultivation using the CSM medium (C/N = 80) to measure the lipid titers and cell growth. As a result, the maximal biomasses of strains po1fk_*ylPYC1*, po1fk_*ylACO1*, and po1fk_*ylMAE1* were slightly decreased compared with po1fk (Figure 2), and deletion of gene *ylIDP2* and *ylPFK* almost does not influence on cell growth. However, cell growth of strains po1fk_*ylPYK* and po1fk_*ylZWF* is severely inhibited. The maximum biomass of po1fk_*ylPYK* and po1fk_*ylZWF* are 72.5% and 52.2% lower than that of the control strain po1fk, respectively, reaching 3.96 and 6.47 (OD_600_). Notably, gene *ylPYK* encodes pyruvate kinase, which converts the reaction of phosphoenolpyruvate to pyruvate in glycolysis. Therefore, deletion of gene *ylPYK* could block the metabolic flux from the glycolytic pathway to TCA cycles. On the other hand, glucose-6-phosphate dehydrogenase ZWF1 catalyzes glucose 6-phosphate to 6-phospho-glucono-1,5-lactone, providing the primary source of cytosolic NADPH in *Y. lipolytica*. It should be noted that NADPH functions as reducing equivalents to maintain cellular redox homeostasis and serves as important electron donors in the anabolic metabolism (Wang et al., 2017). In this study, we found that deletion of gene *ylZWF* gave a 76.9% reduction (0.18 ± 0.05 g/L with the yield of 13.01 mg/g _Glucose_) in the lipid synthesis, indicating that the OxPP pathway is the primary NADPH source for lipogenesis. However, an interesting phenomenon to note is that the surface wrinkle of po1fk_*ylZWF1* is disappeared. We speculated that the reduced surface area of po1fk_*ylZWF1* colony may be attributed to the less oxygen demand for the ZWF1 deficient strain.

Moreover, the lipid titer of po1fk_*ylFAA1*, po1fk_*ylPYC1*, po1fk_*ylACO1*, and po1fk_*ylMAE1* are 0.49 ± 0.11, 0.59 ± 0.10, 0.62 ± 0.02, and 0.67 ± 0.02 g/L, decreased by 38.0%, 25.3%, 21.5%, and 15.2% relatively to po1fk (0.79 ± 0.10 g/L), respectively, suggesting that genes *ylFAA1*, *ylPYC1*, *ylAC O 1*, and *ylMAE* are necessary for the lipid synthesis in *Y. lipolytica*. However, the deletion of gene *ylIDH2* increased the lipid titer, reaching .87 g/L with the yield of 44.8 mg/g _Glucose_, which were 1.10-fold and 1.19-fold of that in the strain po1fk, respectively. It is incomprehensible because the mitochondrial NAD^+^ isocitrate dehydrogenase IDH2 is important for TCA cycle, catalyzing isocitrate to 2-oxoglutarate. To explain this result, we performed a KEGG pathway analysis and found that the mitochondrial NAD^+^ isocitrate dehydrogenases have two isoenzymes in *Y. lipolytica*, encoded by genes *YALI0E05137g* and *YALI0D06303g*, respectively. Therefore, we speculate that deletion of IDH2 downregulated the activity of mitochondrial NAD^+^ isocitrate dehydrogenases, which strengthened the accumulation of citrate to promote the synthesis of lipids. Besides, the deletion of carbon catabolite repressor gene *ylSNF1* significantly increased the lipid titer, reaching 2.45 ± 0.02 g/L with the yield of 100.1 mg/g _Glucose_, which was 3.10-fold and 2.65-fold of that of po1fk, respectively. This result is consistent with the previous research (Seip et al., 2013). The regulator SNF1 is involved in the transition from the growth phase to the oleaginous phase, and allows strains to accumulate lipids even in the nitrogen-abundant medium (Wang et al., 2020). However, how SNF1 regulates the lipid synthesis pathway has not been resolved (Lazar et al., 2018), possibly due to the SNF1-mediated MAP kinase, which phosphorylates acetyl-CoA carboxylase and downregulates lipid synthesis. In brief summary, knocking out mitochondrial IDH2 and SNF1 will improve lipid accumulation in *Y. lipolytica* under the culture condition of using the CSM medium.

### 3.3 Using acetate as carbon source for lipid synthesis by engineered *Y. lipolytica* strains

The acetate uptake pathway in *Y. lipolytica* could function as an acetyl-CoA shortcut, which has been demonstrated to achieve metabolic optimality in producing polyketides (Liu et al., 2019b). Therefore, we turned to use acetate as the carbon source for lipid synthesis. Specifically, extracellular acetate is first catalyzed by acetyl-coA synthase to generate acetyl-coA, which enters into cellular metabolic pathways for maintaining cell activities. However, it should be noted that partial acetyl-CoA would pass through gluconeogenesis into the OxPP pathway to provide NADPH for fatty acid synthesis.

**FIGURE 3:**
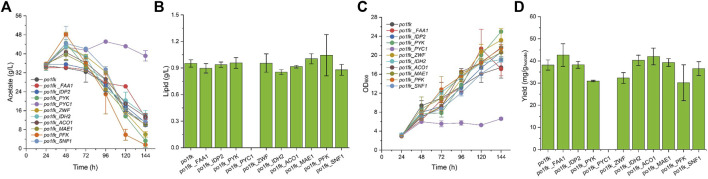
Time profiles of cell growth, acetate consumption, lipid titer and lipid yield in CSM-Acetate medium. **(A)** Glucose consumption of po1fk, po1fk_ylPYK, po1fk_ylACO1, po1fk_ylPFK, po1fk_ylSNF1, po1fk_ylIDP2, po1fk_ylZWF, po1fk_ylIDH2, po1fk_ylMAE, po1fk_ylPYC1, and po1fk_ylFAA; **(B)** Lipid titer of po1fk, po1fk_ylPYK, po1fk_ylACO1, po1fk_ylPFK, po1fk_ylSNF1, po1fk_ylIDP2, po1fk_ylZWF, po1fk_ylIDH2, po1fk_ylMAE, po1fk_ylPYC1, and po1fk_ylFAA; **(C)** Cell growth of po1fk, po1fk_ylPYK, po1fk_ylACO1, po1fk_ylPFK, po1fk_ylSNF1, po1fk_ylIDP2, po1fk_ylZWF, po1fk_ylIDH2, po1fk_ylMAE, po1fk_ylPYC1, and po1fk_ylFAA; **(D)** Lipid yield of po1fk, po1fk_ylPYK, po1fk_ylACO1, po1fk_ylPFK, po1fk_ylSNF1, po1fk_ylIDP2, po1fk_ylZWF, po1fk_ylIDH2, po1fk_ylMAE, po1fk_ylPYC1, and po1fk_ylFAA.

Noticeably, the cultivation pH will keep increasing when sodium acetate is used as a carbon source. For this, an *in situ* pH indicator (bromocresol purple) was used to track the change of cultivation pH (Liu et al., 2019a). By performing the shaking flask, we found that po1fk_*ylPYK*, po1fk_*ylZWF, and* po1fk_*ylFAA1* showed the remarkable recovery of cell growth (Figure 3) when acetate was used as sole carbon source, reaching 20.02 ± 1.18, 20.72 ± 1.56, and 21.38 ± 4.11 (the maximum biomass, OD_600_), respectively. These results indicate that the evolutionary robustness of cells that enable them adapt to alternate carbon sources when central metabolism is compromised, and most importantly, acetate could be used as a shortcut nutrient to support cell growth. However, the cell growth of po1fk_*ylPYC1* was repressed in the CSM-acetate medium, and its maximum biomass (OD_600_) was 5.72 ± 0.50, which could be attributed to the fact that PYC is important for cell to replenish gluconeogenesis when the cells were grown on the acetate media. The maximal lipid titer was produced by po1fk_*ylPFK*, reaching 1.04 ± 0.23 g/L, which is 1.09-fold of that of po1fk (0.95 ± 0.04 g/L). Interestingly, the lipid titer of po1fk_*ylSNF1* was close to that of po1fk using acetate as carbon source. Studies have reported that downregulation of Snf1 can activate the activity of acetyl-CoA carboxylase ACC1, which could facilitate the flux from acetyl-CoA to malonyl-CoA (Gross et al., 2019). Therefore, we speculated the increase flux from acetyl-CoA to malonyl-CoA in the engineering po1fk_*ylSNF1* strain leads to a reduction of NADPH supply and limits the synthesis of lipid under the acetate cultivation.

### 3.4 The effect of overexpressing the selected genes on cell growth and lipid synthesis

**FIGURE 4:**
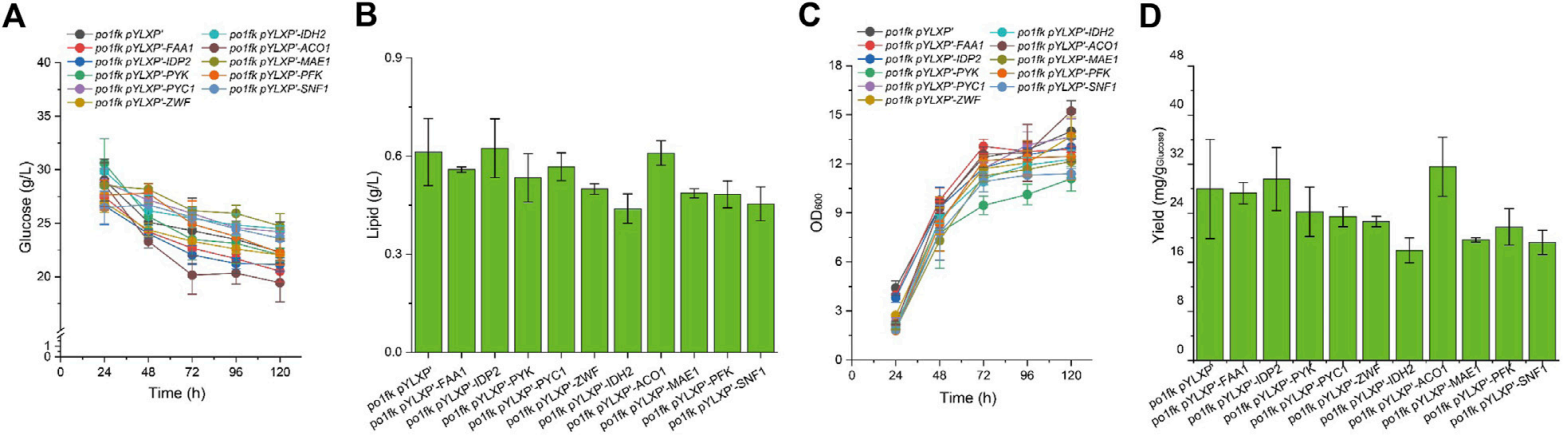
Time profiles of cell growth, glucose consumption, lipid titer and lipid yield in CSM-leu medium. **(A)** Glucose consumption of po1fk pYLXP’, po1fk pYLXP’-ylPYK, po1fk pYLXP’-ylACO1, po1fk pYLXP’-ylPFK, po1fk pYLXP’-ylSNF1, po1fk pYLXP’-ylIDP2, po1fk pYLXP’-ylZWF, po1fk pYLXP’-ylIDH2, po1fk pYLXP’-ylMAE, po1fk pYLXP’-ylPYC1, and po1fk pYLXP’-ylFAA; **(B)** The lipid titer of po1fk pYLXP’, po1fk pYLXP’-ylPYK, po1fk pYLXP’-ylACO1, po1fk pYLXP’-ylPFK, po1fk pYLXP’-ylSNF1, po1fk pYLXP’-ylIDP2, po1fk pYLXP’-ylZWF, po1fk pYLXP’-ylIDH2, po1fk pYLXP’-ylMAE, po1fk pYLXP’-ylPYC1, and po1fk pYLXP’-ylFAA; **(C)** Cell growth of po1fk pYLXP’, po1fk pYLXP’-ylPYK, po1fk pYLXP’-ylACO1, po1fk pYLXP’-ylPFK, po1fk pYLXP’-ylSNF1, po1fk pYLXP’-ylIDP2, po1fk pYLXP’-ylZWF, po1fk pYLXP’-ylIDH2, po1fk pYLXP’-ylMAE, po1fk pYLXP’-ylPYC1, and po1fk pYLXP’-ylFAA; **(D)** Lipid yield of po1fk pYLXP’, po1fk pYLXP’-ylPYK, po1fk pYLXP’-ylACO1, po1fk pYLXP’-ylPFK, po1fk pYLXP’-ylSNF1, po1fk pYLXP’-ylIDP2, po1fk pYLXP’-ylZWF, po1fk pYLXP’-ylIDH2, po1fk pYLXP’-ylMAE, po1fk pYLXP’-ylPYC1, and po1fk pYLXP’-ylFAA.

To further identify the potential functions of the selected genes, we overexpressed these genes under the control of strong constitutive pTEF-intron promoter by the plasmid pYLXP’. Shake flask cultivation of these engineering strains, including po1kf pYLXP’-*ylPFK*, po1kf pYLXP’-*ylPYK*, po1kf pYLXP’-*ylPYC1*, po1kf pYLXP’-*ylMAE1*, po1kf pYLXP’-*ylIDH2*, po1kf pYLXP’-*ylIDP2*, po1kf pYLXP’-*ylFAA1*, po1kf pYLXP’-*ylACO1*, and po1kf pYLXP’-*ylSNF1*, show no significant differences in cell growth (Figure 4). Further, we analyzed the lipid titer of these engineering strains. As shown in Figure 4, individual overexpression of gene *IDP2* result in a significant increase in the lipid titer, reaching 0.62 g/L with the yield of 29.58 mg/g _Glucose_. In *Y. lipolytica*, gene *IDP2* encodes the cytosolic NADP^+^ isocitrate dehydrogenase IDP2, which catalyzes the reaction of isocitrate to 2-oxoglutarate with generating one molecule of NADPH. However, the cytoplasmic TCA cycle in *Y. lipolytica* is incomplete, which lacks cytoplasmic aconitate hydratase, succinyl-CoA ligase, fumarate hydratase*,* etc. Thus, we speculated that has a trans-mitochondrial isocitrate-α-oxoglutarate NADPH shuttle in *Y. lipolytica*, which can be responsible for maintaining the redox homeostasis and transporting the reducing equivalent from mitochondria NADH to cytoplasm NADPH.

## 4 Discussions

In *Y. lipolytica*, lipogenic pathway is consisting of three parts: acetyl-CoA, NAPDH, and lipid metabolism pathways. Herein, we systematically investigated the knockout of ten important genes in these three parts, including *ylPFK*, *ylPYK*, *ylAC O 1*, *ylMAE1*, *ylIDH2*, *ylIDP2*, *ylZWF1*, *ylPYC1*, *ylFAA1*, and *ylSNF1*. Noticeably, pyruvate kinase PYK plays an important role in the glycolysis pathway, which provides the precursor pyruvate for TCA. Deleting gene *ylPYK* resulted in the block of the glycolytic pathway, and thus leaded to an impaired glucose uptake, retarded cell growth and compromised lipid synthesis. Specifically, the previous effort has showed that *Y. lipolytica* possesses strong acetate utilization pathway, which is equivalent or even superior to the hexose utilization pathway (Liu et al., 2019b). Therefore, it is believable that strain po1fk_*ylPYK* showed a remarkable recovery of cell growth using acetate as the carbon source. Specifically, we deduced the overall stoichiometry of glucose or acetate conversion to lipid (Supplementary Note 1). As suggested by the stoichiometry, the yield of lipid synthesized from acetate (0.294 g/g _Acetate_) is higher than that from glucose (0.271 g/g _Glucose_), indicating that acetate is a dominant carbon source for lipid synthesis. As a result, strain po1fk_*ylPYK* produced 0.96 ± 0.06 g/L of lipid using acetate as the carbon source.

The glucose-6-phosphate dehydrogenase ZWF is a key enzyme that catalyzes glucose 6-phosphate to 6-phosphogluconolactone with generating NADPH. NADPH provides reducing power for synthesizing biological macromolecules (such as lipids, proteins, and glycogen) and directly affects cell growth, protein expression, and other secondary metabolites synthesis (polyketides or triterpenoids) (Xu et al., 2018). We found that deletion of PYC1 or MAE1 both decreased the synthesis of lipid, suggesting the POM cycle participates in lipogenesis for NADPH supplementation in *Y. lipolytica*. However, in the CSM-acetate medium, the cell growth of po1fk_*ylPYC1* was repressed, suggesting that the deficiency of pyruvate carboxylase PYC1 would lead to the disruption of gluconeogenesis. Moreover, it is reported that the distributions of the cellular reducing equivalent are highly compartmentalized in eukaryotes, which is attributed to the specific localization of metabolic pathways and impermeabilities of organelles membranes (Kory et al., 2020). Specifically, mitochondria are important organelles for the NADH metabolism due to its respiratory functions, the existence of electron transport chain for oxidative phosphorylation. Therefore, the dynamic balance of the reducing equivalent between mitochondria and cytoplasm are necessary for maintaining cellular redox homeostasis (Spinelli and Haigis, 2018). Our results of deletion of gene *IDH2* and overexpression of gene *IDP2* suggested the existence of a trans-mitochondrial isocitrate-α-oxoglutarate NADPH shuttle in *Y. lipolytica*. Specifically, according to the ways of NADPH supplement, we also deduced the overall stoichiometry of glucose or acetate conversion to lipid and calculated the yield of lipid (Supplementary Note 1), which could help us to understand the influences of NADPH supplement on lipid synthsis.

In addition, protein kinase ylSNF1 is a crucial regulator of glucose signal transduction, which is a negative regulator of the fatty acid biosynthesis pathway and acetyl-CoA carboxylase. It has been identified that SNF1 plays a vital role in the transition from growth to oil production in *Y. lipolytica*. Herein, deletion of genes *ylSNF1* significantly increased the lipid titer in the CSM-leu medium, reaching 2.45 ± 0.02 g/L with the yield of 100.0 mg/g _Glucose_ and the productivity of 0.289 g _Lipid_/L/g _DCW_ (Table2), which is consistent with the previous research (Seip et al., 2013).

**TABLE 2:** The productivity of engineering strains in the different cultivations.

Names	Productivity (g _Lipid_/L/g _DCW_		Names	Productivity (g _Lipid_/L/g _DCW_
	Glucose	Acetate		Glucose
po1fk	0.169	0.158	po1fk pYLXP’	0.125
po1fk_*ylPFK*	0.132	0.139	po1fk pYLXP’-*ylPFK*	0.111
po1fk_*ylPYK*	—/	0.109	po1fk pYLXP’-*ylPYK*	0.137
po1fk_*ylACO1*	0.141	0.121	po1fk pYLXP’-*ylACO1*	0.114
po1fk_*ylMAE1*	0.154	0.139	po1fk pYLXP’-*ylMAE1*	0.114
po1fk_*ylIDH2*	0.177	0.128	po1fk pYLXP’-*ylIDH2*	0.102
po1fk_*ylIDP2*	0.169	0.130	po1fk pYLXP’-*ylIDP2*	0.137
po1fk_*ylZWF1*	0.041	0.118	po1fk pYLXP’-*ylZWF1*	0.104
po1fk_*ylPYC1*	0.136	—/	po1fk pYLXP’-*ylPYC1*	0.119
po1fk_*ylFAA1*	0.144	0.150	po1fk pYLXP’-*ylFAA1*	0.124
po1fk_*ylSNF1*	0.289	0.131	po1fk pYLXP’-*ylSNF1*	0.114

## 5 Conclusion

In this work, we established ten engineered strains with deleting the important gene involved in the central carbon, NADPH, and lipid metabolism, and systematically investigated the influence of such gene deletions on the lipid synthesis and cell growth. As a result, our study demonstrated that NADPH sources for lipogenesis include the OxPP pathway, POM cycle, and isocitrate-2-oxoglutarate shuttle in *Y. lipolytica*. Moreover, the knockout of mitochondrial isocitrate dehydrogenase IDH1 and the carbon catabolite repressor SNF1 could facilitate the lipid synthesis. Besides, acetate is a more favorable carbon source for the lipid synthesis when glycolysis step is impaired, indicating this could be alternate carbon source that reach metabolic optimality in lipid synthesis. This systematic investigation of gene deletions and overexpression across various lipogenic pathways would help us better understand lipogenesis and engineer yeast factories to upgrade the lipid biomanufacturing platform.

## Data Availability

The original contributions presented in the study are included in the article/Supplementary Material, further inquiries can be directed to the corresponding authors.
