# Supplementary material for: Knocking out central metabolism genes to identify new targets and alternating substrates to improve lipid synthesis in Y. lipolytica
Source: Front Bioeng Biotechnol. 2023 Jan 13;11:1098116. doi: 10.3389/fbioe.2023.1098116 (PMC9880266; doi:10.3389/fbioe.2023.1098116)
Supplement: Supplementary file 1 [file DataSheet1.pdf]

Supplementary materials for

Knocking out central metabolism genes to identify new targets and alternating substrates to improve lipid synthesis in *Y. lipolytica*

Jiang Zhu[1], Yang Gu[1,2,*], Yijing Yan[1], Jingbo Ma[2,5], Xiaoman Sun[1], Peng Xu[2,3,4,*]

[1] School of Food Science and Pharmaceutical Engineering, Nanjing Normal University, Nanjing, China

[2] Department of Chemical, Biochemical and Environmental Engineering, University of Maryland, Baltimore County, Baltimore, MD, USA

[3] Department of Chemical Engineering, Guangdong Technion-Israel Institute of Technology (GTIIT), Shantou, Guangdong, China

[4] The Wolfson Department of Chemical Engineering, Technion-Israel Institute of Technology, Haifa, Israel

[5] College of Biological and Pharmaceutical Engineering, West Anhui University, Lu'an, China

*Corresponding author, guyang@nnu.edu.cn (GY) and pengxu@gtiit.edu.cn (PX), Tel: (86)-754-88077163

Table S1 Primers used in this study

Names	Sequences
ylZwf_UpF	cctaaatttgatgaaagcctaggcaaaaggggcatattggaatgccg
ylZwf_UpR	tgtatgctatacgaagttatggtgatgtatgtatggtgagtgtcttgtg
ylZwf_DwF	gctagcgagacaataacggaggagtattcaaacaatttgttccagtcaagaaact
ylZwf_DwR	atccttttatcagacatagtcgacctcggacactgaagagtcgaccg
ylZwf_DwChkR	agcaaacaaagtcgccaagaagaaatc
ylZwf_UpChkF	ccctacaaagcgtcgcactg
ylMAE1_UpF	gcatccctaaatttgatgaaagcctaggcctttagctcaaatctcgcatttgagactc
ylMAE1_UpR	ataatgtatgctatacgaagttatggcgaatatcagcgtgtacaggagt
ylMAE1_DwF	gagacaataacggaggagtcgacagcgtggaatagtggaatacagctt
ylMAE1_DwR	atccttttatcagacataggaactgtttttggttttgcgacg
ylMAE1_DwChR	cgggtgaaaaggccgttgttg
ylMAE1_UpChkF	gagagaacattattgccaatctgtcgtc
ylPYC1_UpF	ggcatccctaaatttgatgaaagcctaggcgcggttagtgcggttctataactc
ylPYC1_UpR	cgtataatgtatgctatacgaagttatggtgtgcgataccgtggtgt
ylPYC1_DwF	gctagcgagacaataacggaggagtcgacttccgatcttatttgcaagattgtgcgg
ylPYC1_DwR	gttacatccttttatcagacataaatgcgccttctcaatcccga
ylPYC1_DwChkR	ggctctaacaattgatagacgttgtacacg
ylPYC1_UpChkF	tccacctgctgtggccatg
ylIDH2_UpF	tccctaaatttgatgaaagcctaggagccgtgcgtgcaattagac
ylIDH2_UpR	tgtatgctatacgaagttattgtgatttctttgggggttccacg
ylIDh2_DwF	gagacaataacggaggagtcgacattatcaagcgactcaagtagacgatataacga
ylIDH2_DwR	tacatccttttatcagacatatgatgtacagttgaggttgacaacagtgc
ylIDH2_DwChkR	tcgctcttccggacggttagc
ylIDH2_UpChkF	agggtaggggtacttgtcctctac
ylFAA1_UpF	tccctaaatttgatgaaagcctaggtcgagggagtggttacgggac
ylFAA1_UpR	ataatgtatgctatacgaagttattctttgttagttgttggaaaaaaaaaggaggg
ylFAA1_DwF	cgagacaataacggaggagtcgacagtgtttaaaccctctgacagttttgca
ylFAA1_DwR	gttacatccttttatcagacatatgtgtctgggtggacctccc
ylFAA1_DwChkR	tcgaaaatccccaagattccgctgtg
ylFAA1_UpChkF	tatgccaatggttgcgcatttccc
ylIDP1_UpF	atccctaaatttgatgaaagcctagggcacgacagggacaaagtggtgc
ylIDP1_UpR	taatgtatgctatacgaagttatggttttagaaagggctagcagccg
ylIDP1_DwF	gcgagacaataacggaggagtcgacaggacctggcttaggggaaaatg
ylIDP1_DwR	ttacatccttttatcagacatagctttgtcacggcttcagcg
ylIDP1_DwChkR	ctttacctcatactgcggctgtt
ylIDP1_UpChkF	tgagtggatgagaagggactgtttgta
ylSNF1_UpF	tggcatccctaaatttgatgaaagcctaggcaagattgagagttggagtggtgg
ylSNF1_UpR	cgtataatgtatgctatacgaagttatttgtgaggtggtggaaggagtgg
ylSNF1_DwF	ctagcgagacaataacggaggagtcgacgcacttgtagagcacactagggat
ylSNF1_DwR	gttacatccttttatcagacatattcttctgcccgaattgcaccag
ylSNF1_DwChkR	gttcgtacctcttttccaaccaaaacaa
ylSNF1_UpChkF	tccagctgaatctgaacgggtttct

ylMCT1_UpF	catccctaaatttgatgaaagcctagggtttctgtcagtcgttgttcctcgataa
ylMCT1_UpR	ataatgtatgctatacgaagttatgttgtggtggtacaactcgcagtatc
ylMCT1_DwF	gctagcgagacaataacggaggagtcgacatggtctgtctggtgagcagc
ylMCT1_DwR	ttacatccttttatcagacatagccccaacactgttgctcttca
ylMCT1_DwChkR	gaaatccggggcggcatttc
ylMCT1_UpChkF	ccacaaccttggcttcccagt
ylDGA1_ChkF	gtttatgcattctgttggaccttagtctg
ylDGA1_ChkR	gttatctaccacgatttttggtttctgaggc
ylDGA2_ChkF	ctatcgccccaaagtgtttctagca
ylDGA2_ChkR	gagatggcatgccaacgttgac
ylDGA2_DwF	gctagcgagacaataacggaggacataacactcatcagtagcctttacagtgat
ylDGA2_DwR	ccttttatcagacatagcggccgcttgctcttgtaattccatagataatatatacgaaa
ylDGA2_UpF	aaatttgatgaaaggcggccgccttgggagtgtatttggaaaatgacttgg
ylDGA2_UpR	tgtatgctatacgaagttattttgcgggcggtacgggtaca
ylDGA1_DwF	gctagcgagacaataacggaggaggaaaactgcctgggttaggcaaat
ylDGA1_DwR	atcagacatagcggccgctctctgatggcctggagcgag
ylDGA1_UpF	aatttgatgaaaggcggccgcatgctgcgggcggatcctgg
ylDGA1_UpR	tgtatgctatacgaagttatagcttttgttttgtgtgacttgtctgt
ylPYK_UpF	ttgatggcatccctaaatttgatgaaagcctaggtgcgcgcttctgttgtttgcaac
ylPYK_UpR	cttcgtataatgtatgctatacgaagttattgtaactgtggtgtgaatttctccgcagg
ylPYK_DwF	caataacggaggagtcgacactttttaacacaaacctattagattataaaacatacagcatg
ylPYK_DwR	tgttacatccttttatcagacataacaaccccaacggagtgtgc
ylPYK_DwChkR	ctccgctgccaccaaaaacg
ylPYK_UpChkF	cacgcatctcttctccccaac
ylPFK_F	cactttttgcagtactaaccgcagATTGAAGGAATCTCCTTTGCGTCGTTTG
ylPFK_R	gacaggccatggaactagtcggtaccCTAACAAGGATCAATAATACCCTGCTCCTTCG
ylSNF1_F	cagcactttttgcagtactaaccgcagGCGACCGAACACGTGGAACAC
ylSNF1_R	caggccatggaactagtcggtaccTTACTTCTCACTCTCCTTCTGAGAACTCACG
ylZWF1_F	gcactttttgcagtactaaccgcagACTGGCACCTTACCCAAGTTCG
ylZWF1_R	caacgtggggacaggccatggaactagtcggtaccTCACGAGGAGCCCTTGGTGAC
ylPYK_F	gcactttttgcagtactaaccgcagATTTACACCGCCAATTCGTCCCC
ylPYK_R	gtggggacaggccatggaactagtcggtaccTTAGACACACTCGAGAACTCGGAGAG
ylPYC1_F	gaccagcactttttgcagtactaaccgcagTCCAACGTTCCTGAGACCAAGGTG
ylPYC1_R	gggacaggccatggaactagtcggtaccTTAAGCCCGCACAATCTTGCAAATAAGAT
ylMAE1_F	cgaccagcactttttgcagtactaaccgcagTTACGACTACGAACCATGCGACCC
ylMAE1_R	cgtggggacaggccatggaactagtcggtaccCTAGTCGTAATCCCGCACATGGATGA
ylIDP2_F	cactttttgcagtactaaccgcagTCCACCACCGCTACTCGAGG
ylIDP2_R	ggggacaggccatggaactagtcggtaccCTAAGCCAGGTCCTTCTTCAGTCTGTTG
ylIDH2_F	cagcactttttgcagtactaaccgcagCTCAACCTTAGAACCGCCCTTCGAG
ylIDH2_R	gggacaggccatggaactagtcggtaccCTACTTGAGTCGCTTGATAATCTGCTCAGC
ylFAA1_F	cactttttgcagtactaaccgcagGTCGGATACACAATTTCCTCAAAGCCC
ylFAA1_R	gggacaggccatggaactagtcggtaccCTAAGACTGCTCGTAGCACTCATCAATTTC
ylACO1_F	gcactttttgcagtactaaccgcagCTGGCTTCTCGAGTTTCCATCAAGG
ylACO1_R	ggggacaggccatggaactagtcggtaccTTATTTCTTGGAGGCAGCCATCTTGTTGAG

TEF_R	tttgatatgtggggagaaggggggct
XPR_F	taacctcccacactcctttgacataacg

Note S1 the theoretical lipid yield of different pathways for the synthesis of lipid

Specifically, the theoretical lipid yield of different pathways for the synthesis of lipid were compared and analyzed.

1.1 De novo fatty acid biosynthesis

1.1.1 Using glucose for fatty acid synthesis

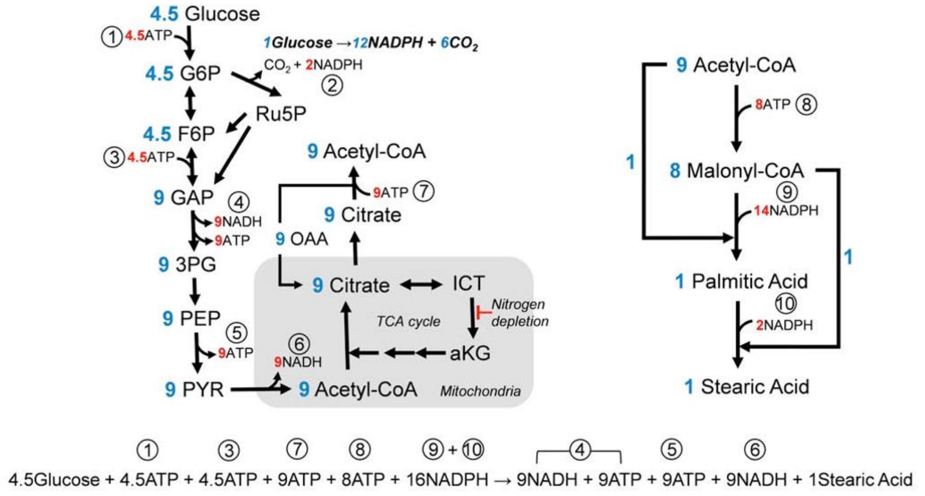

Thus, the overall stoichiometry of fatty acid (stearic acid) biosynthesis is:

4.5 Glucose + 8 ATP +16 NADPH → 1 Stearic Acid + (9In+9Ex) NADH

Specifically, the pentose phosphate pathway supplies all NADPH. In case of redox deficient pathways, reducing equivalents are assumed to be generated via oxidation of substrate completely to CO_2 via the pentose phosphate pathway leading to 12 NADPH per glucose molecule oxidized. Therefore, the production of 1 molecule of stearic acid requiring 5.83 molecule of glucose (4.5 + 16/12 = 5.83). As a result, the yield of de novo fatty acid biosynthesis from glucose is 0.271 g/g glucose.

1.1.2 Using acetate for fatty acid synthesis

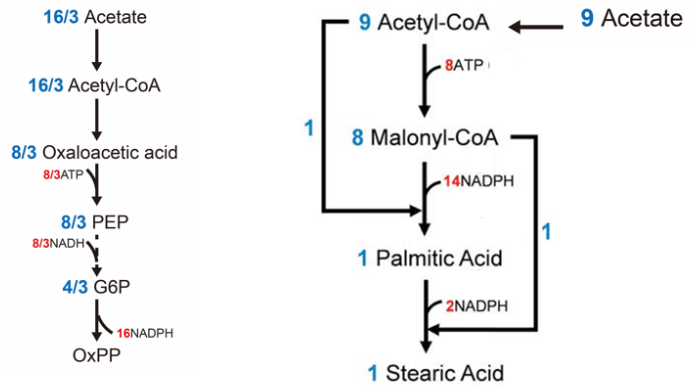

(16/3+9)Acetate + 8/3ATP + 8/3NADH + 8ATP+16NADPH → 1 Stearic Acid+ 16NADPH

Thus, the overall stoichiometry of fatty acid (stearic acid) biosynthesis is:

(16/3+9)Acetate + (8/3+8)ATP + 8/3NADH → 1 Stearic Acid

Specifically, the extracellular acetate enters into the cellular metabolism by the action of transporters. Then, acetate is converted to acetyl-CoA by acetyl-CoA synthetase (ACS). Further, acetyl-CoA is converted into glucose 6-phosphate by gluconeogenesis, and enters into the oxidative pentose phosphate pathway (OxPP) to generated NADPH. In addition, acetyl-CoA can be oxidized to generate ATP or NADH by the TCA cycle, and the stoichiometry is

$$\text{Acetyl-CoA} + 3\ NAD^+ + FAD + GDP + 3\ H_2O \rightarrow 3\ NADH + FADH_2 + GTP + 2\ CO_2$$

Therefore, in case of redox deficient pathways, ATP or NADH are assumed to be generated via oxidation of substrate completely to CO_2 via the TCA cycle leading to 10 ATP (3*2.5+1*1.5+1=10) or 4 NADH (one molecule of $FADH_2$ and GTP can be transformed into one molecule of NADH) per acetate molecule oxidized. Therefore, the production of 1 molecule of stearic acid requiring 16.1 molecule of acetate (16/3 + 9 + 3.2/3+ 2/3 = 16.1). As a result, the yield of de novo fatty acid biosynthesis from acetate is 0.294 g/g acetate.

1.2 The POM pathway

1.2.1 Using glucose for fatty acid synthesis

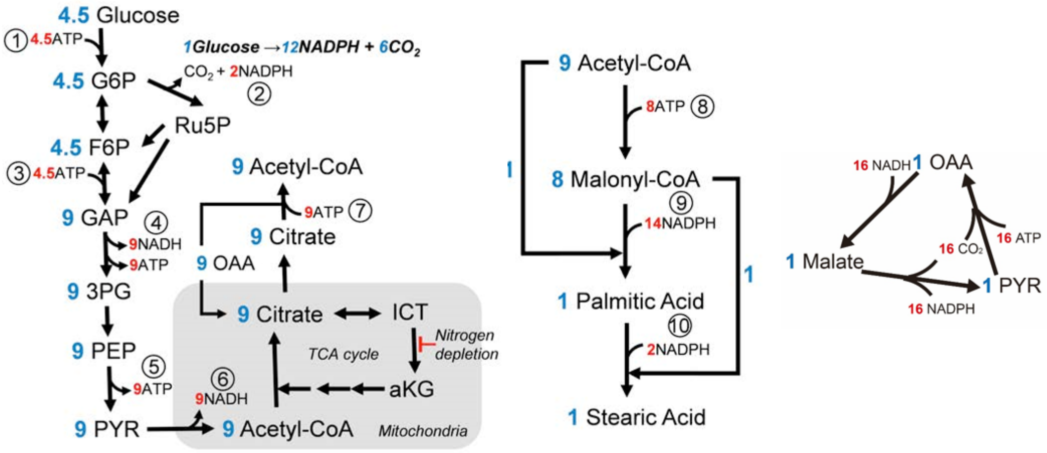

$$4.5\text{Glucose} + 8\text{ATP} + 16\text{NADPH} + 16_{Ex}\text{NADH} + 16\ \text{ATP} \rightarrow 1\ \text{Stearic Acid} + (9_{In}+9_{Ex})\ \text{NADH} + 16\text{NADPH}$$

Thus, the overall stoichiometry of fatty acid (stearic acid) biosynthesis is:

$$4.5\text{Glucose} + 24\text{ATP} + 7_{Ex}\text{NADH} \rightarrow 1\ \text{Stearic Acid} + 9_{In}\ \text{NADH}$$

Specifically, it needs extra 19 molecules of ATP, which will consume 19/30 molecules of glucose. Therefore, the production of 1 molecule of stearic acid requiring 5.13 molecule of glucose (4.5 + 1.9/3= 5.13). As a result, the yield of de novo fatty acid biosynthesis from glucose is 0.307 g/g glucose by using the POM pathway.

1.2.2 Using acetate for fatty acid synthesis

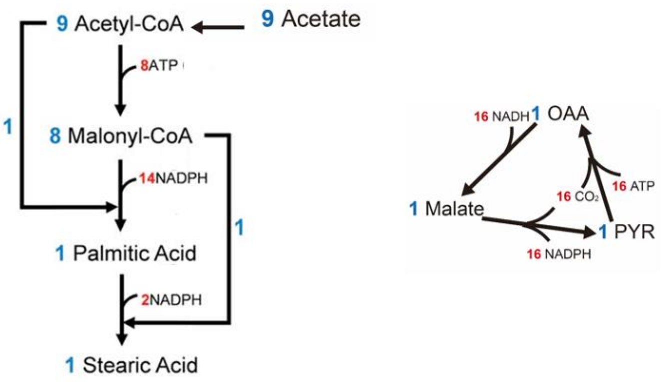

9Acetate + (8+16)ATP + 16NADH +16NADPH → 1 Stearic Acid +16NADPH

Thus, the overall stoichiometry of fatty acid (stearic acid) biosynthesis is:

9Acetate + 24ATP + 16NADH → 1 Stearic Acid

Here, acetyl-CoA can be oxidized to generate ATP or NADH by the TCA cycle, and the stoichiometry is

Acetyl-CoA + 3 NAD^+ + FAD + GDP + 3 H_2O →3 NADH + $FADH_2$ + GTP + 2 CO_2

Therefore, the production of 1 molecule of stearic acid requiring 15.4 molecule of acetate (9 + 2.4 +16/4 = 15.4). As a result, the yield of de novo fatty acid biosynthesis from acetate is 0.307 g/g acetate by using the POM pathway.

1.3 The trans-mitochondrial isocitrate-α-oxoglutarate NADPH shuttle

1.3.1 Using glucose for fatty acid synthesis

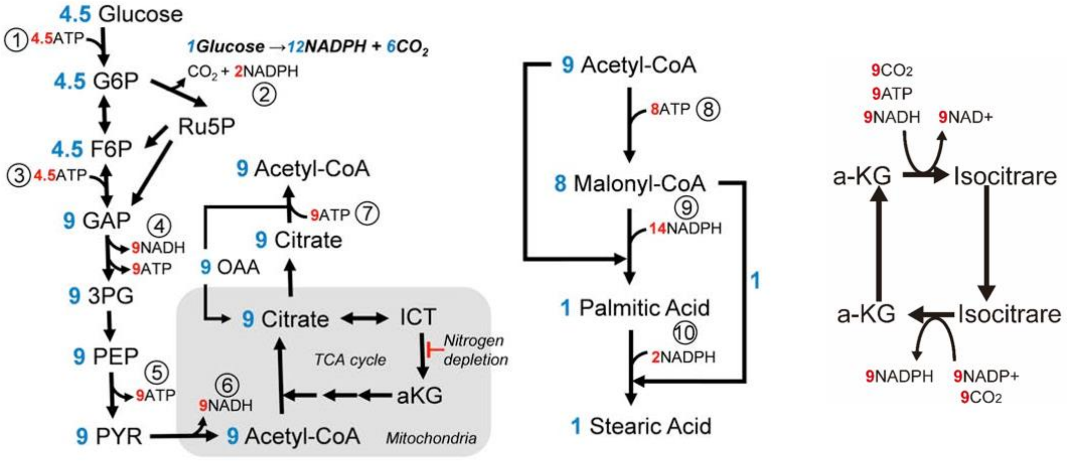

4.5Glucose + 8ATP + 16NADPH + 16_{In}NADH + 16ATP→ 1 Stearic Acid + (9_{In}+9_{Ex}) NADH + 16NADPH

Thus, the overall stoichiometry of fatty acid (stearic acid) biosynthesis is:

4.5Glucose + 24ATP + 7_{In}NADH→ 1 Stearic Acid + 9_{Ex} NADH

Specifically, it needs extra 19 molecules of ATP, which will consume 19/30 molecules of glucose. Therefore, the production of 1 molecule of stearic acid requiring 5.13 molecule of glucose (4.5 + 19/30= 5.13). As a result, the yield of de novo fatty acid biosynthesis from glucose is 0.307 g/g glucose by using the POM pathway.

1.3.2 Using acetate for fatty acid synthesis

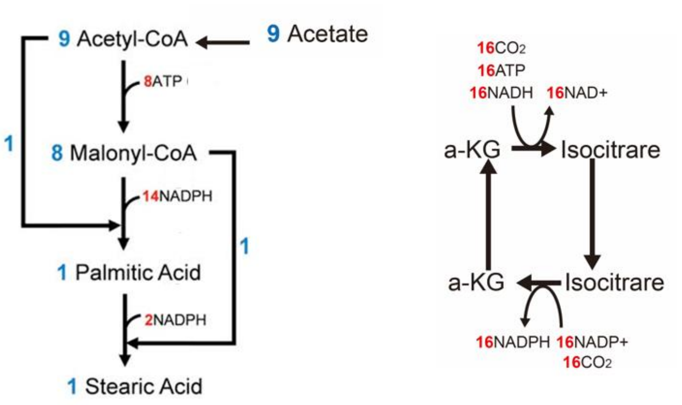

$$9\text{Acetate} + (8+16)\text{ATP} + 16\text{NADH} + 16\text{NADPH} \rightarrow 1 \text{ Stearic Acid} + 16\text{NADPH}$$

Thus, the overall stoichiometry of fatty acid (stearic acid) biosynthesis is:

$$9\text{Acetate} + 24\text{ATP} + 16\text{NADH} \rightarrow 1 \text{ Stearic Acid}$$

Here, acetyl-CoA can be oxidized to generate ATP or NADH by the TCA cycle, and the stoichiometry is

$$\text{Acetyl-CoA} + 3\ NAD^{+} + \text{FAD} + \text{GDP} + 3\ H_2O \rightarrow 3\ \text{NADH} + FADH_2 + \text{GTP} + 2\ CO_2$$

Therefore, the production of 1 molecule of stearic acid requiring 15.4 molecule of acetate (9 + 2.4 +16/4 = 15.4). As a result, the yield of de novo fatty acid biosynthesis from acetate is 0.307 g/g acetate by using the POM pathway.
